# Contribution of cryopreservation to the cumulative live birth rate: a large multicentric cycle-based data analysis from the Italian National Registry

**DOI:** 10.1007/s10815-019-01566-y

**Published:** 2019-08-28

**Authors:** G. Scaravelli, P. E. Levi-Setti, C. Livi, G. La Sala, F. M. Ubaldi, E. Greco, M. E. Coccia, A. Borini, A. Revelli, G. Ricci, V. Vigiliano, R. De Luca, S. Bolli, L. Rienzi, Collaborators, R. Spoletini, A. Drovanti, F. Benini, M. T. Villani, L. Albricci, M. T. Varricchio, V. Basile, M. Cattoli, A. Nizzi, K. Skerk, L. Speziale, S. Fiaccavento

**Affiliations:** 1 ART Italian National Register, National Centre for Diseases Prevention and Health Promotion, National Health Institute, Rome, Italy; 2 IRCCS, Division of Gynecology and Reproductive Medicine, Humanitas Clinical and Research Institute, Rozzano, Milan, Italy; 3 Department of Obstetrics, Gynecology and Reproductive Science, School of Medicine, Yale University, New Haven, CT USA; 4 ART Center DEMETRA, Florence, Italy; 5 Department of Obstetrics and Gynecology, Arcispedale S. Maria Nuova, Reggio Emilia, Italy; 6 University of Modena and Reggio Emilia, Modena, Italy; 7 GENERA Centre for Reproductive Medicine, Clinica Valle Giulia, Via de Notaris 2B, Rome, Italy; 8 Center for Reproductive Medicine, European Hospital, Via Portuense 700, 00149 Rome, Italy; 9 DAI-MI –AOU, Careggi-University of Florence, Florence, Italy; 10 9.baby, Family and Fertility Center, Tecnobios Procreazione, Bologna, Italy; 11 Gynecology and Obstetrics 1U, Physiopathology of Reproduction and IVF Unit, Sant’Anna Hospital, University of Torino, Torino, Italy; 12 Institute for Maternal and Child Health, IRCCS Burlo Garofolo, Trieste, Italy; 13 Department of Medicine, Surgery and Health Sciences, University of Trieste, Trieste, Italy

**Keywords:** IVF, ICSI, Live birth, Cumulative delivery rate, Cryopreservation, National Register

## Abstract

**Purpose:**

To estimate the contribution of cryopreservation to the cumulative live birth rate (CLBR) after law modification in Italy in the era of vitrification and freeze-all.

**Methods:**

The Italian National Registry performed a cycle-based data collection. Nine Italian IVF clinics were involved incorporating a total of 10,260 fresh cycles performed between January 2015 and April 2016 resulting in 9273 oocyte retrievals and 3266 subsequent warming cycles from the same oocyte retrievals performed up to December 2016. Mean female age was 37 ± 4.3 years. Primary outcome measure was CLBR per oocyte retrieval. Confounding factors were tested in multivariate regression analysis, and the relative impact of cryopreservation to the CLBR in different patient categories was calculated.

**Results:**

CLBR per oocyte retrieval was 32.6%, 26.5%, 18.7%, 13.0%, and 5.5% for women younger than 36, aged 36–39, 40–41, and older than 41 years, respectively. The total relative contribution of oocyte/embryo cryopreservation was 40.6% (95% CI 38.41–42.75). An association between maternal age, number of oocytes retrieved, fertilization rate, cryopreservation, and cumulative live birth was shown. When adjusted for confounders, a 2.3-fold increase was observed in the chance of live birth when cryopreservation was performed (OR 2.3; 95% CI 1.99–2.56). In high responder patients (> 15 oocytes retrieved) where freeze-all was applied in 67.6% of cycles to avoid the risk of hyper stimulation syndrome, the relative contribution of vitrification to the CLBR was 80.6%.

**Conclusions:**

Cryopreservation is essential in IVF and should always be available to patients to optimize success rates. Multicentric, cycle-based data analyses are crucial to provide infertile couples, clinicians, and regulatory bodies with accurate information on IVF effectiveness including fresh and cryopreserved cycles.

## Introduction

The Italian Registry of ART is responsible by law to retrospectively collect baseline information and IVF outcomes of all treatment cycles performed in Italy since 2004 (Repubblica Italiana, Law 40/2004). Only summary data were collected up until now, with clear limitations in data analysis. The cumulative chance of success, including fresh and cryopreserved cycles, cannot for instance be calculated if cycle-based data are not available. In turn, infertile couples, clinicians, and regulatory bodies cannot have a real picture of the effectiveness of IVF treatments across the country. These aspects are particularly important in those countries where law limitations are in force. For instance, in Italy, embryo cryopreservation was strictly forbidden from 2004 and 2009, when only oocyte cryopreservation could be applied.^1^–^4^ Since 2009, embryo cryopreservation can be performed with the aim to protect women’s health (Corte Costituzionale 2009)^5^, ^6^ (i.e., to minimize the risks of ovarian hyperstimulation syndrome and/or multiple pregnancy and to optimize the chance of success according to medical indication). To be able to calculate the impact of this change in regulations on a patient’s chance of success, CLBR including fresh and cryopreserved cycles need to be carefully estimated.

The aim of this study is to calculate the contribution of cryopreservation to the CLBR in different patient populations by analysis of cycle-based data collected from 9 representative Italian IVF clinics.

## Materials and methods

### Source of data

In January 2016, new software was developed by the Italian National Registry allowing individual data collection from single IVF treatments based on the same items that were previously collected in a cumulative form. The software was then implemented in December 2016 in 9 voluntary representative Italian IVF clinics for the number of cycles performed annually and type of service (public and private) (Table 1). Variables were validated based on the experience of a Lombardy county pilot data collection program of single IVF treatments (Assisted Reproductive Technology Lombardia Network).

**Table 1:** ART centers involved in the study

ART centers	Type of service	City/region	All fresh IVF cycles 2015–2016		Fresh IVF retrievals included in the study	
			*N*	% of the total number of cycles	*N*	% of the total number of retrievals included
Centro Medicina della Riproduzione - AOU città della Salute e della Scienza di Torino - Ospedale Sant’Anna	Public	Torino/Piemonte	901	4.8	528	5.7
IRCCS Istituto Clinico Humanitas - Dipartimento di Ginecologia e Medicina della Riproduzione	Private covered by NHS	Milano/Lombardia	4.875	26.0	2.586	27.9
SSD Procreazione Medicalmente Assistita - IRCCS Burlo Garofalo	Public	Trieste/Friuli Venezia Giulia	694	3.7	385	4.2
Centro per la Diagnosi e la Terapia della Sterilità Involontaria di Coppia “P-Bertocchi”- A.O. Arcispedale *S. Maria* Nuova	Public	Reggio Emilia/Emilia Romagna	2.425	12.9	1.223	13.2
9.baby - Family and Fertility Center, Tecnobios Procreazione, Bologna	Private	Bologna/Emilia Romagna	1.245	6.6	595	6.4
SOD di Procreazione Medicalmente Assistita - Università degli Studi di Firenze - A.O. Careggi	Public	Firenze/Toscana	1.321	7.0	577	6.2
Centro di Procreazione Assistita “Demetra”	Private covered by NHS	Firenze/Toscana	3.226	17.2	1.628	17.6
Medicina e Biologia della Riproduzione - European Hospital	Private	Roma/Lazio	1.546	8.2	754	8.1
GENERA - Clinica Valle Giulia - Casa di cura - SPA	Private	Roma/Lazio	2.540	13.5	997	10.8

**Fig. 1:**
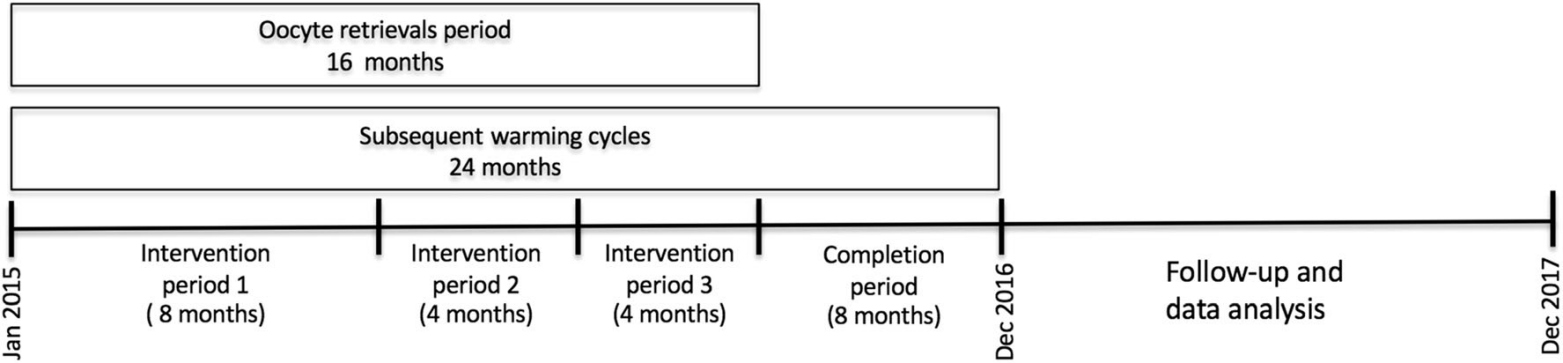
Study design. Intervention period = period during which oocyte retrievals have been performed. Completion period = period following oocyte retrievals where warming cycles are performed. Period 1 = patients that performed intervention in the first 8 months, followed up by a completion period of 16 months; period 2 = patients that performed intervention between 8 and 12 months from the beginning of the study period, followed up by a completion period of 12 months; period 3 = patients that performed intervention between 12 and 16 months from the beginning of the observation period, followed up by a completion period of 8 months

Data were collected retrospectively for all consecutive autologous IVF cycles undertaken between January 2015 and December 2016. For the purpose of the analysis, 10,260 fresh cycles performed between 1 January 2015 and 30 April 2016 resulting in 9273 oocyte retrievals and 3266 subsequent warming cycles from the same oocyte retrievals performed up to December 2016 were included (Fig.1 study design).

Only one fresh IVF cycle per patient was included. Oocyte retrieval cycles that were performed for fertility preservation purposes and donor cycles were excluded.

### Variables

*N* = 102 variables for each cycle were collected, including patient and cycle characteristics. In particular, maternal age, paternal age, cause of infertility, ovarian stimulation protocol, source of gametes, sperm quality, number of collected, mature and inseminated oocytes, fertilized oocytes, embryos and blastocysts obtained, transferred and cryopreserved, pregnancy, miscarriage, delivery, and neonatal outcomes (birth weight and weeks of gestation) were collected. The primary outcome measure was cumulative live birth rate (CLBR) defined as the number of deliveries with at least one live birth resulting from one oocyte retrieval cycle, including all cycles in which fresh and/or frozen embryos were transferred, until one delivery with a live birth occurred or until all embryos were used, whichever occurred first. The delivery of a singleton, twin, or other multiples was registered as one delivery.^7^, ^8^

### Regulation

Briefly, in Italy, no restriction exists with respect to the ovarian stimulation protocol to adopt and/or to the number of embryos to be transferred simultaneously. The number of oocytes to be inseminated is not anymore defined by law but has to be decided and certified by the clinician according to the best clinical practice and the estimated chance of pregnancy for each patient. Of note, all the viable embryos produced must be either transferred or cryopreserved, regardless of their morphological/genetic quality, and they cannot be discarded or donated for research purposes. Preimplantation genetic testing is possible also for fertile couples. Surrogacy is not allowed.

### Statistical analysis

Univariate and multivariate logistic regressions were performed to evaluate associations with CLBR. The model included the following patient and cycle characteristics: maternal age, paternal age, cause of infertility, ovarian stimulation protocol, source of spermatozoa, sperm quality, number of collected, mature and inseminated oocytes, type of insemination procedure (IVF vs ICSI), fertilized oocytes, cryopreservation, and IVF center. We tested all two-way interactions between pairs of predictors included in our multivariate analyses and used a Bonferroni correction (for multiple testing) *p* value threshold of 0.05 to define statistical evidence of an interaction. The predictive value of the resulting model was assessed by calculating the area under the curve of receiver operator characteristics (AUROC). To evaluate the level of agreement between the estimated and the observed probabilities (calibration), the Hosmer-Lemeshow test was used. All statistical analyses were performed using IBM SPSS Statistics 23.

## Results

The overall CLBR was 21.7% per oocyte retrieval (95% CI 20.88–22.57). Mean female age was 37 ± 4.3 years. The patient population was distributed as follow: 34.6% younger than 36, 32.9% aged 36–39, 17.3% 40–41, and 15.2% older than 41 years. Causes of infertility were tubal (11.3%), anovulatory (2.9%), endometriosis (3.9%), male (26.1%), combination of known causes (23.3%), and unknown cause (32.6%).

The intervention period in this study was 24 months, 16 months for oocyte retrievals and a minimum of 8 months for subsequent cryo-cycles (completion period). To understand the time needed to complete an IVF cycle, three different periods were analyzed: period 1 = patients that performed intervention in the first 8 months, followed up by a completion period of 16 months; period 2 = patients that performed intervention between 8 and 12 months from the beginning of the study period, followed up by a completion period of 12 months; period 3 = patients that performed intervention between 12 and 16 months from the beginning of the observation period, followed up by a completion period of 8 months (Fig. 1).

A total of 8240 cycles (89.9% CI 89.3–90.5) were concluded at the time of the analysis, with either a delivery or no supernumerary available oocytes or embryos still cryopreserved. When analyzing treatment discontinuation (no pregnancy obtained but cryopreserved embryos or oocytes still available), according to the completion period, we observed that 13.9%, 11.9%, and 9.9% of cycles were not concluded during the periods of 8 months, 12 months, and 16 months following oocyte retrievals, respectively (Table 2).

**Table 2:** Discontinuation according to the completion period starting from oocyte retrieval date (see Fig. 1). Discontinuation of the treatment = cycle not concluded (no pregnancy obtained but cryopreserved embryos/oocytes still available). Completion period = period following oocyte retrieval date where warming cycles are performed

Completion period								
Intervention period 1 considering 16 months for completion			Intervention period 1 + 2 considering 12 months for completion			Intervention period 1 + 2 + 3 considering 8 months for completion		
*N* cycles	Discontinuation of the treatment	% discontinuation of the treatment	*N* cycles	Discontinuation of the treatment	% discontinuation of the treatment	*N* cycles	Discontinuation of the treatment	% discontinuation of the treatment
4.936	487	9.9	7.028	836	11.9	9.273	1.286	13.9

**Fig. 2:**
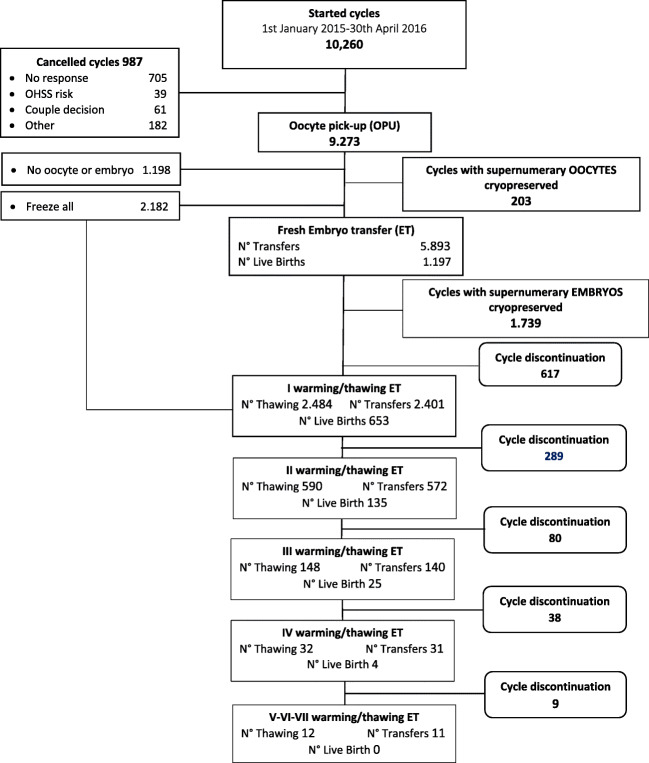
Flow-chart of the study. Discontinuation represents the number of patients that had not completed their treatment with oocytes and/or embryos still cryopreserved and no live birth obtained

A total of 1197 LB were obtained after fresh embryo transfers (12.9% per oocyte retrieval and 20.3% per transfer). A total of 483 oocytes, 817 embryos, and 1563 blastocysts obtained from the same oocyte retrieval cycles were warmed. Survival rates were 77.3%, 85.7%, and 96.4%; respectively. An additional 817 deliveries were obtained after warming embryo transfers (25.0% per cycle and 25.9% per transfer) (Fig.2). A total of 40.6% of all live births included in the CLBR were a result of cryopreservation. In particular, oocyte cryopreservation contributed only 0.3% (6) instead of transfer of thawed embryos that contributed 40.3% (811). The multivariate analysis showed an association between patient (maternal age), cycle (number of oocytes retrieved, fertilization rate, and cryopreservation) characteristics, and CLBR per oocyte retrieval (Table 3). The predictive value of the model was moderate with AUCROC = 0.75 (*p* < 0.001).

**Table 3:** Logistic regression analysis adjusted for significant patients and cycle confounders

Characteristic	Categories	Univariable odds ratio of live birth (95% CI)	Multivariable^a^ odds ratio of live birth (95% CI)	*p* value^b^
Maternal age (years)	≤ 35	1	1	< 0.001
	36–37	0.743 (0.647–0.853)	0.793 (0.685–0.918)	
	38–39	0.478 (0.413–0.553)	0.59 (0.506–0.688)	
	40–41	0.31 (0.263–0.365)	0.433 (0.365–0.515)	
	≥ 42	0.119 (0.094–0.152)	0.189 (0.148–0.243)	
Retrieved oocytes	≤ 3	1	1	< 0.001
	4–6	2.923 (2.38–3.59)	1.751 (1.413–2.171)	
	7–10	4.589 (3.762–5.598)	2.12 (1.712–2.626)	
	11–15	6.919 (5.64–8.489)	2.627 (2.095–3.295)	
	> 15	8.878 (7.175–10.985)	2.679 (2.105–3.41)	
Fertilization rate	< 50%	1	1	< 0.001
	50–70%	2.263 (1.876–2.731)	1.8 (1.477–2.193)	
	> 70%	3.286 (2.762–3.91)	2.378 (1.974–2.865)	
Cryopreservation	No	1	1	< 0.001
	Yes	4.466 (4.019–4.963)	2.256 (1.986–2.563)	

^a^Multivariable adjusted = mutual adjustment for uncorrelated significant variables listed in column 1

^b^*p* value for multivariable association

**Fig. 3:**
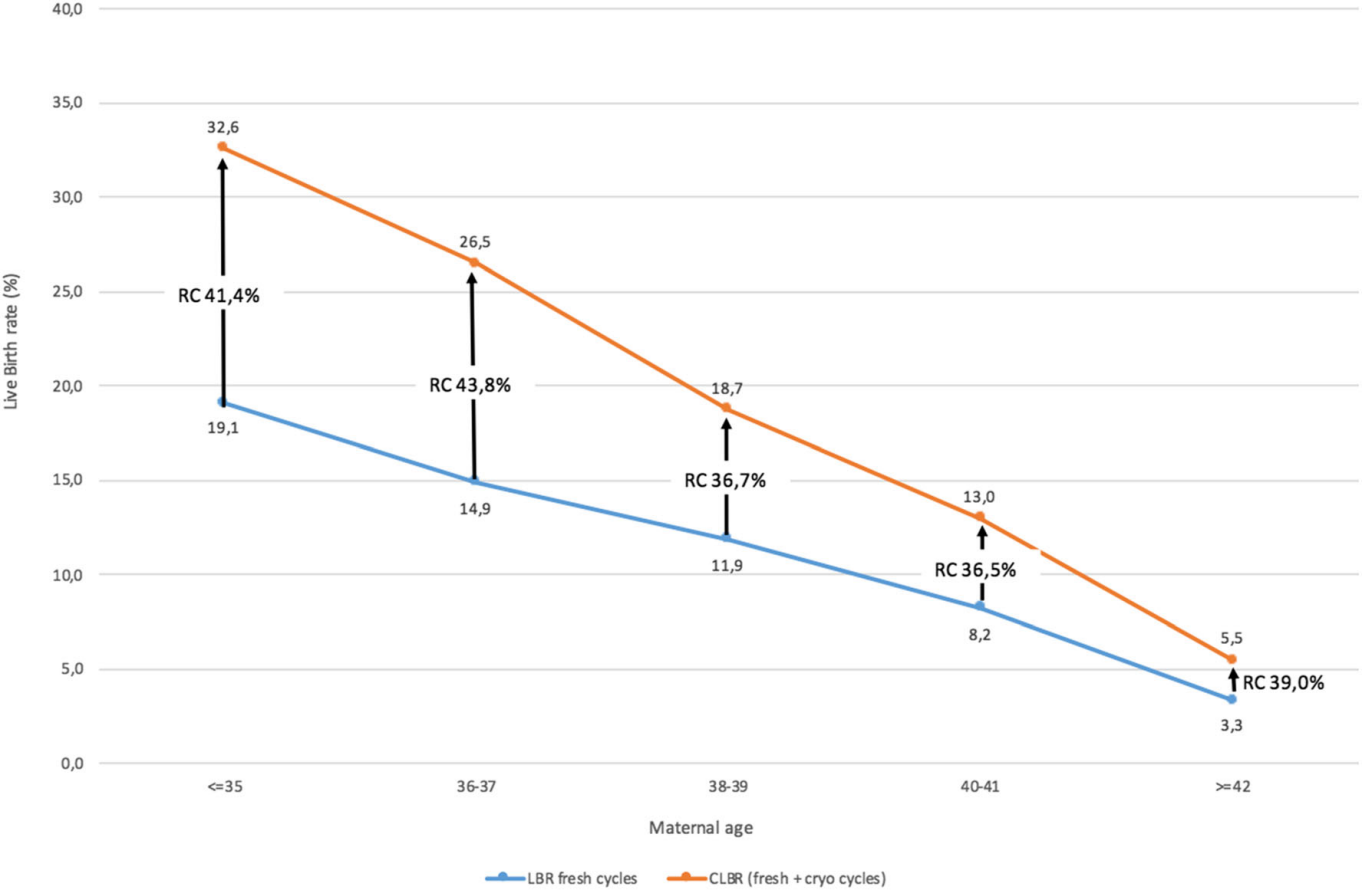
Live birth rate (LBR) per fresh cycle and cumulative LBR (CLBR) including fresh and warming cycles according to female age

As expected, the probability of live birth significantly decreased with increasing maternal age. However, cryopreservation significantly contributed to the success rate in all patient populations (the relative increase in LBR was 41.4% for women younger than 36, 43.8% aged 36–39, 36.7% 40–41, and 39.0% for those older than 41 years) (Fig. 3).

**Fig. 4:**
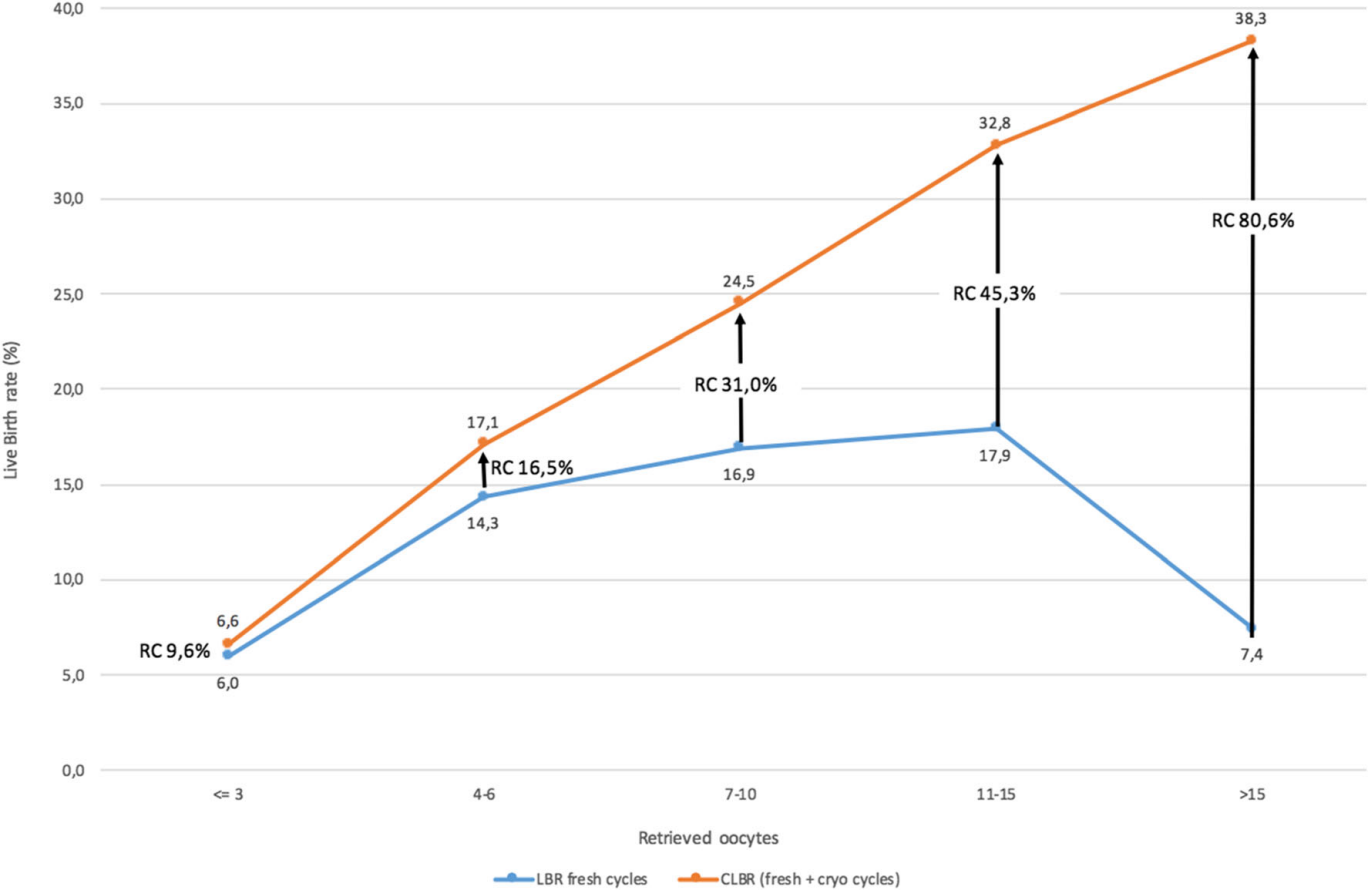
Live birth rate (LBR) per fresh cycle and cumulative LBR (CLBR) including fresh and warming cycles according to the number of oocytes retrieved

Increasing the number of oocytes retrieved resulted in higher chance of success. Poor responders (< 3 oocytes) demonstrated a significantly lower CLBR vs suboptimal responders (4–6 oocytes) (OR 1.7; 95% CI 1.41–2.17), normal responders (7–10 oocytes) (OR 2.1; 95% CI 1.71–2.63), good responders (11–15 oocytes) (OR 2.6; 95% CI 2.09–3.29), and high responders (> 15 oocytes) (OR 2.7; 95% CI 2.10–3.41). It emerged that the more oocytes were available, the greater the chance to obtain a live birth when fresh and cryo-cycles were included. In particular, the relative contribution of cryopreservation to the CLBR increased in relation to the ovarian response (9.6%, 16.5%, 31.0%, 45.3%, and 80.6% for poor, suboptimal, normal, good, and high responders, respectively) (Fig. 4).

The use of ICSI was not significantly associated with the CLBR (univariate OR = 1.1 and 95% CI 0.92–1.22). However, a high fertilization rate (FR) increased the chance of success independently from the insemination technique used and the number of oocytes retrieved (when FR > 70%, OR = 2.4, and 95% CI 1.97–2.86). This could be attributed to the higher number of embryos available for transfer and cryopreservation and thus to the higher relative contribution of cryopreservation to the CLBR (25% vs 43%, for bad and good FR, respectively).

We observed the freeze-all approach to be efficient and safe with no impact on the CLBR (univariate OR = 1.7 and 95% CI 1.53–1.93 while multivariate OR = NS). Ovarian hyperstimulation syndrome (OHSS) was not reported when freeze-all was adopted to avoid this complication (*N* = 924; 9.0% of cycles), while 12 cases (0.13%) of severe OHSS were identified after fresh embryo transfers. Thromboembolic events were not reported. It is important to note that in the high responder population (> 15 oocytes retrieved), the relative impact of cryopreservation was as high as 80.6% due to greater application of the freeze-all strategy aimed at increasing cycle safety (67.6% of oocyte retrievals).

## Discussion

National Registries have the responsibility to provide reliable reporting of IVF outcomes. The primary concern in the estimation of IVF success rate is the overall chance of at least one delivery including both fresh and cryopreserved oocyte/embryo transfers from the same oocyte retrieval. A second important challenge is to inform patients, clinicians, and regulatory bodies about safety issues by clearly reporting the risks associated with the technology and clinical management.

The Italian National Registry publishes age-stratified outcome rates annually. Summary data collection, however, can only separately calculate fresh and cryopreserved cycle outcomes while cumulative success rates can only be estimated. Due to new advances in cryopreservation since the introduction of vitrification into IVF laboratories (reviewed by^9^), fresh cycles alone are poorly representative of the overall success rate.^10^ The present study, performed on cycle-based data collection, shows for the first time in Italy the real contribution of cryopreservation to the CLBR in the new era of vitrification and freeze-all. Our results confirm that the success rate is clearly underestimated when only fresh transfers are considered, i.e., in the young patient population, the live birth rate per oocyte retrieval was 19.1% with fresh ET which increased up to 32.6% when cryopreserved cycles were included; while in high responder patients, the live birth rate increased from 7.4 to 38.3% when cryo-cycles were included (relative increase of 80.6%). This latter figure is also due to the large application of freeze-all aimed at reducing the risk of hyperstimulation. Overall, it must be emphasized that embryo cryopreservation was the main contributor to increased CLBR compared with oocyte cryopreservation. In our study, more than 1 pregnancy out of 3 was in fact obtained using cryopreserved embryos. This finding underlines the importance of this technology in IVF that should be available to all patients to maximize their chance of success.

When embryo cryopreservation was allowed in Italy, it resulted in a sudden application of this technology in spite of the well-consolidated practice of cryopreserving oocytes. There is a plethora of reasons that boosted such a change in the clinical strategy. First of all was cost-benefit. Specifically, whenever the reason for cryopreservation was not fertility preservation for medical or non-medical reasons,^11^ but when it was performed as part of a conventional IVF cycle, cryopreserving embryos as opposed to oocytes ensured a lower workload for the laboratories (single insemination procedure, less embryos as compared with oocytes to be cryopreserved, and higher cryo-survival rates, especially when dealing with blastocysts). Moreover, observation of fertilization and early preimplantation development allows assessment of embryo developmental competence before short- or even long-term storage is performed. Beyond strategic and logistical concerns, technical reasons also supported the switch from oocyte to embryo cryopreservation. Specifically, the oocyte is the largest cell in humans, and its shape, the amount of water it contains, and the permeability of its membrane make it more fragile to all the cryopreservation-induced stresses, thereby limiting the efficacy of such clinical practice. Indeed, higher survival rates after cryopreservation have been consistently reported for embryos compared with oocytes, especially when vitrification protocols are adopted.^9^

To perform an analysis of CLBR that includes collectively fresh and thawed cycles resulting from each oocyte collection, a completion period following oocyte retrievals is necessary. Our data show that 16 months of interventions (oocyte retrievals) followed by a minimum of 8 months of follow-up (thawing cycles) are needed to obtain full information on 88.9% of oocyte retrievals (treatment discontinuation = 11.1%). Even when a 16-month period follow-up was possible for warming cycles (oocyte retrievals performed in the first 8-month period of the study), 9.9% of cycles were still not concluded. Considering the whole study period, 1 patient out of 3 among those not pregnant after the fresh embryo transfer that had supernumerary cryopreserved oocytes/embryos did not come back for their first warming cycle. The reasons that induce patients to postpone or not perform their cryo-cycles need further investigation. This could be partly due to a lack of information about the real potential of cryopreserved embryos to contribute to the success rate. Another possibility is that couples move from one center to another before completing their cycle. A better follow-up will be possible only when cycle-based data analysis is implemented in all IVF centers and a unique identification number is assigned to each individual couple.

Our study reinforces previous findings related to the identification of variables affecting IVF success rates.^12^–^18^ Besides the obvious negative influence of maternal age, other factors were also independently predictive of CLBR in our cycle-based database. Here we assessed the contribution of cryopreservation in association with specific patient and cycle characteristics.

Our data confirm the clear positive relationship between the numbers of oocytes available for insemination and CLBR. Patients should thus be informed that the higher the oocyte yield, the higher the probability of achieving a delivery, when both fresh and cryo-cycles are considered. Moreover, our data also endorse the evidence that a high response to ovarian stimulation, resulting in more than 15 oocytes retrieved, does not impair the CLBR but, on the contrary, increases the chance of success when subsequent warming cycles are included. This finding is consistent with previous recent reports based on CLBR.^19^, ^20^

The use of ICSI was previously shown to be an effective treatment for male factor infertility.^14^, ^16^, ^18^ Our data confirm that when ICSI is consistently used (84.7% of cycles in this study), male factor infertility is not a negative predictor of CLBR. However, we found that a low fertilization rate (independent of the insemination method) was associated with a decreased chance to obtain a live birth because the contribution of cryopreservation was smaller in these cases. We suggest that this parameter should be included as a confounder of IVF success for future analysis.

In our analysis, freeze-all had no negative impact on the CLBR. The new advances in cryobiology, with the introduction of vitrification for both oocyte and embryo cryopreservation, have now made this strategy routinely available to clinics. The aims are to reduce the risks related to ART technology (namely OHSS) and/or to extend the time for embryo evaluation. New prospects for increasing the safety and efficiency of ART technology are thus expected in the near future with the systematic use of cryopreservation.^9^, ^21^, ^22^

We believe that the outcomes of this study can considerably help IVF centers to improve their results by adopting the best strategies for clinical/embryological management. Principally, ovarian stimulation strategies should be aimed at obtaining the maximum number of oocytes, freeze-all approach should be adopted to minimize the occurrence of OHSS without impacting the overall results, and finally clinical management should focus on encouraging oocyte/embryo-warming cycles to improve CLBR. The access to and optimization of cryopreservation programs are in fact essential factors to ensure quality and safety of treatments. It is important to note that the mean female age in our patient population was 37 ± 4.3 years (with 65.4% of women over 35 years old). This is in line with previous reports that have shown that the Italian population undergoing IVF is particularly old compared with the rest of Europe (De Geyter et al. 2018)^23^. However, the relative contribution of cryopreservation was significant in all age groups (> 35%) further underlining the importance of this technology also for poor prognosis patients.

This study also shows that the potential of cycle-based data analysis to assess CLBR and IVF Registers and Centers should be encouraged to proceed with this approach.

## Conclusions

Cycle-based data collection allows accurate analysis of cumulative IVF outcomes to be performed, by identifying factors that can affect the success of this procedure and those that may induce adverse outcomes. Cryopreservation has become a key factor allowing live birth rates in different patient populations to be substantially improved. These analyses aim to facilitate patient counseling, clinical decision-making, and informing regulatory bodies about advancement of the technology.
